# Gonococcal outer membrane vesicle vaccines: bacterial population biology, clinical trials, immune profiling, and vaccine design

**DOI:** 10.1038/s41541-026-01410-2

**Published:** 2026-03-05

**Authors:** Zhenyi Gu, Anastasia Unitt, Odile B. Harrison, Jeremy P. Derrick, Ann E. Jerse, Samantha A. McKeand, Christoph M. Tang

**Affiliations:** 1 Sir William Dunn School of Pathology, University of Oxford, Oxford, UK; 2 Department of Biology, University of Oxford, Oxford, UK; 3 Lydia Becker Institute of Immunology and Inflammation, School of Biological Sciences, Faculty of Biology, Medicine and Health, The University of Manchester, Manchester, UK; 4 Department of Microbiology and Immunology, Uniformed Services University, Bethesda, MD USA

**Keywords:** Computational biology and bioinformatics, Diseases, Immunology, Microbiology

## Abstract

Gonorrhoea is a global health concern exacerbated by rising antimicrobial resistance. Retrospective analyses indicate that outer membrane vesicle (OMV) vaccines derived from *Neisseria meningitidis* (MeNZB, 4CMenB) may offer partial cross-protection against gonococcal infection. This review outlines the influence of gonococcal population biology on coverage, immune responses elicited by 4CMenB, and emerging strategies that offer the prospect of rationally designed vaccines dedicated to the prevention of gonococcal disease.

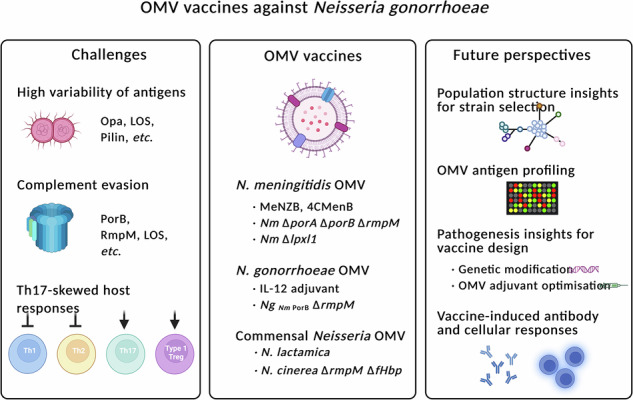

## Introduction

Gonorrhoea is a sexually transmitted infection (STI) caused by *Neisseria gonorrhoeae* (the gonococcus), a Gram-negative diplococcus that colonises the human genital tract^1^. Gonorrhoea is a global public health concern, with an estimated 82.4 million new infections reported worldwide in 2020^2^. Untreated, gonorrhoea can lead to severe health complications, including epididymitis, pelvic inflammatory disease and infertility^1^. Emerging antimicrobial resistance (AMR) is therefore a significant concern^3^, as the control of infection is currently largely based on the successful treatment of infected individuals and their contacts. As a result, there is an urgent need to develop safe and effective vaccines to prevent the spread of *N. gonorrhoeae*. Efforts to develop vaccines targeting *N. gonorrhoeae* have been hampered, however, by the remarkable ability of the bacterium to avoid clearance by the human immune system. This has been attributed to the extensive diversity in surface-exposed antigens, as well as its capacity to manipulate the innate and adaptive immune systems^4^.

Recently, the MeNZB vaccine and subsequently the licenced vaccine 4CMenB (Bexsero, GSK) have gained attention as potential candidates for preventing gonococcal infections. Both vaccines contain outer membrane vesicles (OMVs) from *Neisseria meningitidis*, a close relative of *N. gonorrhoeae*. In 2017, a retrospective case-control study reported 31% vaccine effectiveness of MeNZB against symptomatic gonococcal infection^5^, with the following studies showing a similar moderate effectiveness for 4CMenB, ranging from 23 to 46%^6–9^. In August 2025, the UK Health Security Agency approved the use of 4CMenB vaccination to prevent gonorrhoea in high-risk populations, including individuals suffering from repeat infections, such as men who have sex with men (MSM)^10^. This programme, together with results from ongoing randomised controlled trials, will inform the development of more targeted OMV vaccines that are specifically designed for *N. gonorrhoeae*.

Here, we provide an overview of current research on OMV-based vaccines and their potential for controlling *N. gonorrhoeae* infection. We examine: (i) the genetic variation of *N. gonorrhoeae*, which must be considered in vaccine development and evaluation, (ii) the immunological responses elicited by OMV vaccines, and (iii) recent advances in OMV vaccine development. Finally, we discuss key challenges and future opportunities in developing effective OMV vaccines against gonorrhoea.

## Genetic diversity of *N. gonorrhoeae*

*N. gonorrhoeae* is naturally competent for DNA uptake^11^, and can therefore readily acquire genetic material from other *N. gonorrhoeae* strains and other species, including those from the *Neisseria* genus. This process, known as horizontal gene transfer (HGT), includes mechanisms such as transformation and conjugation. Much of the AMR observed in *N. gonorrhoeae* arises from HGT of chromosomally-encoded gene fragments associated with AMR (e.g. mosaic *penA*) or through conjugation of plasmid-mediated resistance genes^12^. HGT therefore facilitates genetic diversity in the gonococcus and plays an important role in conferring AMR.

Genetic diversity can also arise as a result of antigenic and phase variation. For example, during human infection, *pilE*, which encodes a major subunit of the type IV pilus, undergoes antigenic variation through gene conversion, leading to changes in the PilE protein, therefore avoiding immune recognition^13^. Phase variation, the reversible ON/OFF switching of gene expression, is often caused by slipped-strand mispairing during replication of repetitive DNA sequences in open reading frames or promoters, and can lead to frameshift mutations. For example, the *opa* genes, which encode opacity-associated proteins involved in adherence, contain CTCTT repeat units that undergo variation in the number of repeats, altering Opa expression^14^. This variation affects the binding of Opa proteins to carcinoembryonic antigen-related cell adhesion molecules (CEACAMs), which are differentially expressed along the female reproductive tract; CEACAM1 predominates in the upper tract, while CEACAM5 is more abundant in the lower tract^15^. Therefore, changes in Opa expression modulate bacterial colonisation in distinct anatomical sites, and these differences further influence host immune responses, including neutrophil-mediated phagocytosis^16^ and CD4^+^ T cell activation^17^. Similar phase variation occurs in lipooligosaccharide (LOS) biosynthesis genes, such as those encoding glycosyl transferases: *lgtA*, *lgtC* and *lgtD* contain homopolymeric G tracts ^18^, while *lgtG* contains poly-C tracts ^19^. The key antigens involved in this review are summarised in Table 1.

**Table 1:** Key *N. gonorrhoeae* antigens

Key antigens for immune evasion		
Antigen	Location	How it contributes to immune evasion
PilE	Outer membrane	PilE is the main pilin in the type IV pilus and undergoes extensive antigenic variation^13^
Opacity-associated protein (Opa; protein II)	Outer membrane	Phase variation of *opa* leads to expression of various Opas, which are key to bacterial adherence and invasion^14^
Lipooligosaccharide (LOS)	Outer membrane	Phase variation of LOS glycosyl transferase (*lgt*) genes leads to diverse LOS structures^18,19^
Porin B (PorB; protein I)	Outer membrane	PorB recruits human complement inhibitors, such as C4b-binding protein^29^ and factor H^30^, and inhibits dendritic cell-stimulated CD4^+^ T cell proliferation^33^
Reduction modifiable protein M (RmpM; protein III)	Periplasm	RmpM induces blocking antibodies which bind to the bacterial surface without bactericidal activity^31^

Key antigens of other functions		
Antigen	Location	Function
β-barrel assembly machinery protein A (BamA)	Outer membrane	Integral outer membrane protein assembly^83^
Immunoglobulin A1 (IgA1) protease	Outer membrane	IgA1 protease cleaves the hinge region of human IgA1^87^
Membrane protein 2	Outer membrane	Membrane protein 2 is a membrane-associated lipoprotein^54^
Methionine transporter Q (MetQ)	Outer membrane	MetQ binds to methionine, essential for nutrition acquisition and highly conserved^83^
Multiple transferrable resistance protein E (MtrE)	Outer membrane	MtrE exports host-derived antimicrobials and increases antimicrobial resistance^83^
* Neisseria* heparin-binding antigen (NHBA)	Outer membrane	NHBA is a recombinant protein in 4CMenB, which is on the surface of *N. gonorrhoeae*^47^
PilQ	Outer membrane	PilQ is an outer membrane secretin for pilus biogenesis (no antigenic variation of *pilQ*)^88^
Transferrin-binding proteins (Tbp)	Outer membrane	TbpA is essential for iron uptake from host transferrin and TbpB increases the efficiency^83^

These mechanisms lead to a *N. gonorrhoeae* population that exhibits high genetic diversity. The population structure of *N. gonorrhoeae* has been investigated using several molecular-based approaches, including multi-locus sequence typing (MLST), which indexes the diversity found at seven housekeeping genes^20^, *N. gonorrhoeae* sequence typing for antimicrobial resistance (NG-STAR), which is based on seven chromosomally-encoded AMR-associated genes and is used to track AMR^21^, and core genome MLST (cgMLST), which clusters *N. gonorrhoeae* genomes using 1668 core genes^22^. Unlike many pathogens, *N. gonorrhoeae* has a fundamentally non-clonal population structure as a result of frequent intraspecies HGT, which causes diversification and reassortment over time. cgMLST approaches, however, improve resolution of the gonococcal population structure, allowing discrete genome lineages to be identified, which can be used to inform the choice of *N. gonorrhoeae* strains for evaluating cross-protective responses and strain coverage, or for generating OMVs.

Recently, a stable *N. gonorrhoeae* genomic lineage nomenclature has been developed based on the barcoding system of Life Identification Number (LIN) codes, which uses a refined cgMLST scheme comprised of 1,430 genes (cgMLST v 2.0). This hierarchical LIN code nomenclature conveys lineage information at multiple levels of resolution within one code, and provides immediate context to an isolate’s ancestry^23^. It provides a robust framework for overlaying variation in surface antigens onto stable genomic lineages and can therefore inform strain selection and antigenic cross-reactivity.

Isolates can also be assessed based on variation in proteins found in OMVs. The GC OMV peptide typing scheme, available on PubMLST (https://pubmlst.org/neisseria)^24^, was generated precisely for this purpose. This scheme comprises 26 proteins, selected based on their predicted cellular localisation and relative abundance in *N. gonorrhoeae* OMVs^25^. Each isolate’s combination of peptide variants defines its OMV peptide type (OMVT), which has been assigned to 7786 *N. gonorrhoeae* isolates in PubMLST (as of June 2025).

**Fig. 1:**
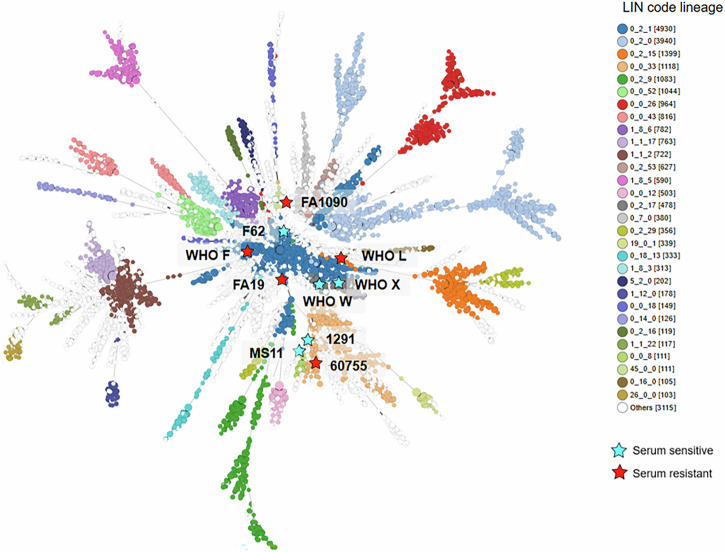
Clustering of *N. gonorrhoeae* based on OMV peptide type. This minimum spanning tree shows the clustering of 7786 *N. gonorrhoeae* isolates that have been assigned an outer membrane vesicle peptide type (OMVT) in PubMLST (https://pubmlst.org/neisseria)^24^. The tree was generated based on allelic profile across 26 OMV-associated loci^25^ and coloured by LIN lineage^23^. To facilitate clearer visualisation in this figure, branch lengths are not in proportion, as true branch lengths were very short. Serum-resistant isolates discussed in this review are marked by red stars, including FA1090^89^, FA19^90^, 60755^77^, WHO F and WHO L^91^. Serum-sensitive isolates are marked by blue stars, including 1291, MS11^89^, F62^90^, WHO X^92^ and WHO W^91^. The tree demonstrates how these laboratory strains fail to represent the full diversity of OMV types sampled from the wider gonococcal population.

Isolates can then be clustered based on their OMVT allelic profiles (Fig. 1). Of note, the OMVT-based clustering reproduces many of the LIN code lineages, indicating a clear association between OMV antigen profiles and the core genome. This suggests that horizontal gene transfer in *N. gonorrhoeae* has not disrupted the linkage between OMV antigenic variation and genomic lineage. Therefore, antigenic and phylogenetic relationships among isolates can be jointly visualised and used to inform strain selection for vaccine development.

Unfortunately, many of the isolates from which OMVs are most studied, including laboratory strains such as MS11, belong to older lineages and may have OMV types that are not representative of the current *N. gonorrhoeae* population. It is apparent that the nine strains discussed in this review fail to capture the majority of the gonococcal diversity shown in Fig. 1. This potential incongruence between the antigens carried by the circulating gonococcal population and laboratory strains could negatively impact the efficacy of vaccines designed using laboratory strain-derived OMVs. Further research is needed to explore this hypothesis.

In summary, *N. gonorrhoeae* exhibits extensive genetic variability driven by HGT, antigenic variation and phase variation. These mechanisms facilitate immune evasion and antimicrobial resistance, allowing the pathogen to persist and reinfect hosts. Tools such as the GC OMV peptide typing scheme and LIN code can be used to visualise the population structure, and to provide a rational basis for selecting strains in vaccine development and evaluation.

## Immune evasion by *N. gonorrhoeae*

*N. gonorrhoeae* is a strict human pathogen, with potential gonococcal infection documented as far back as ancient Egypt around 1550 BC^26^. Through its long and close association with the human immune system, *N. gonorrhoeae* has evolved to avoid clearance by innate and adaptive responses. These adaptations facilitate persistent colonisation of the urogenital tract and contribute to the failure of infected individuals to develop protective immunity.

The complement system is an integral part of both innate and adaptive immunity. It promotes serum-mediated killing of *N. gonorrhoeae* through the formation of the membrane attack complex, which is composed of complement components C5 through to C9. The membrane attack complex forms a pore in the outer membrane of Gram-negative bacteria and leads to cell lysis^27^. However, at cervical mucosal surfaces, *N. gonorrhoeae* turns complement to its advantage. Cervical epithelial cells produce C3, which is activated to C3b. C3b deposits on bacteria and is rapidly converted to iC3b. The iC3b-opsonised *N. gonorrhoeae* then binds to CR3 (CD11b/CD18) on cervical epithelial cells, allowing adherence to and invasion of host cells^28^.

Apart from exploiting complement, *N. gonorrhoeae* expresses several surface molecules that interfere with complement activation. These include the porin PorB, reduction-modifiable protein M (RmpM), and LOS (Table 1). PorB binds human complement inhibitors such as C4b-binding protein^29^ and factor H^30^, hindering the classical and alternative complement pathways, respectively. RmpM is closely associated with PorB, and can induce blocking antibodies i.e. antibodies that fail to activate complement while preventing the binding of bactericidal antibodies to other surface antigens^31^. Moreover, the addition of host-derived sialic acid to bacterial LOS enables *N. gonorrhoeae* to mimic host glycosphingolipids, facilitating immune evasion through molecular mimicry^32^.

*N. gonorrhoeae* also manipulates host cellular immune responses. Studies of cellular immunity have shown that individual gonococcal antigens inhibit CD4^+^ T cell responses, with PorB inhibiting mouse dendritic cell-stimulated CD4^+^ T cell proliferation^33^ and Opa suppressing activation of primary human CD4^+^ T cells via CEACAM1^34^. In the host, upon pathogen recognition, antigen-presenting cells produce cytokines that programme the differentiation of CD4^+^ T cells into various subsets such as T helper (Th)1, Th2, Th17 and type 1 regulatory T (Treg) cells^35^. Th1 responses, driven by interleukin (IL)-12, promote interferon gamma (IFN-γ) production, whereas Th17 responses, induced by IL-6, lead to IL-17 production and local recruitment of neutrophils, and Treg cells produce IL-10 to maintain local immunosuppression^35^. Th17-skewed responses have been observed in serum, urine and cervicovaginal secretions from infected individuals with elevated levels of IL-6 or IL-17^36–39^. This drives inflammation at the mucosal surface through recruitment of neutrophils.

**Fig. 2:**
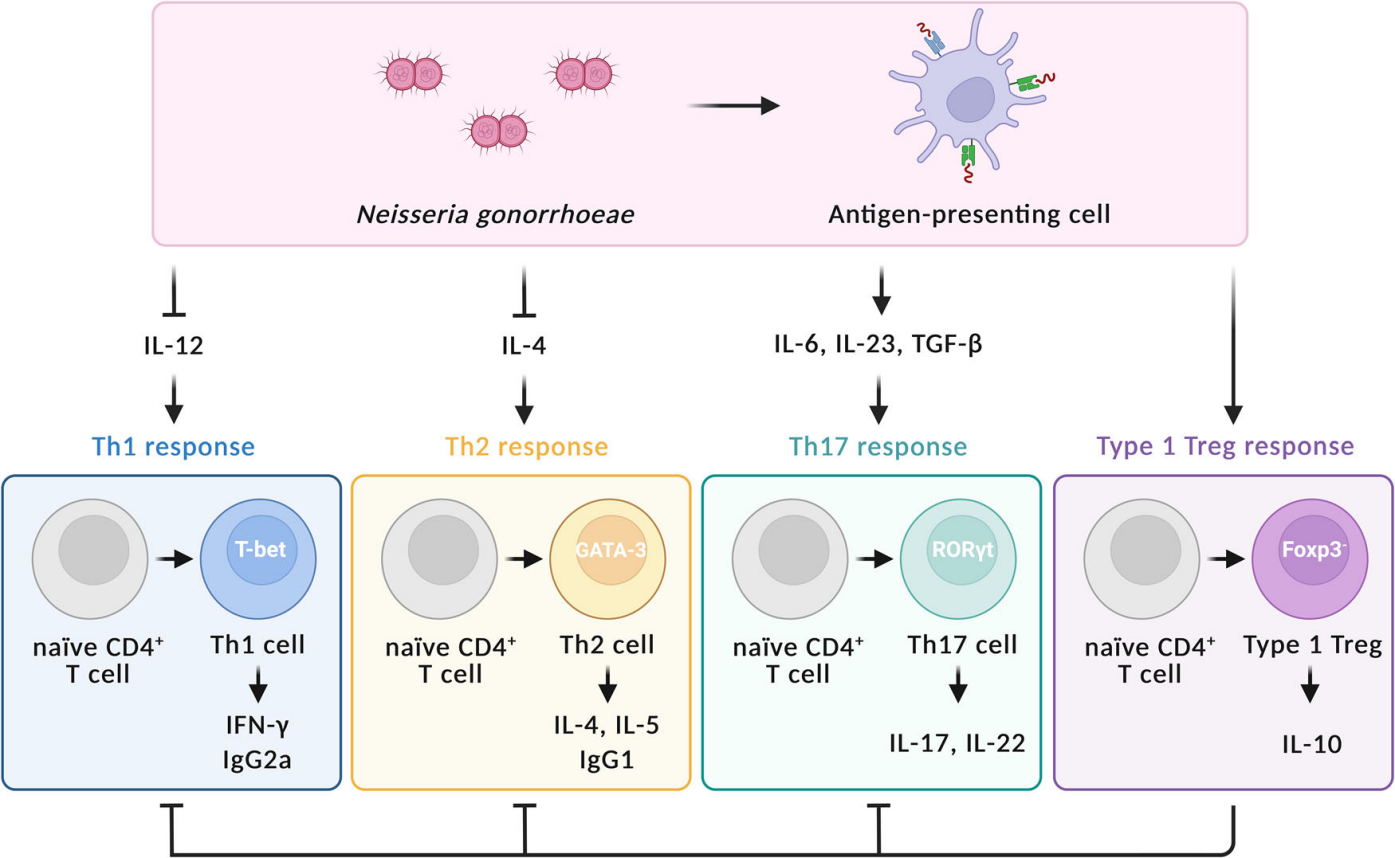
Murine T cell responses induced by *Neisseria gonorrhoeae.* Upon stimulation by *N. gonorrhoeae*, antigen-presenting cells secrete cytokines including interleukin (IL)-6, IL-23 and transforming growth factor (TGF)-β, which differentiate naïve CD4^+^ T cells into T helper (Th)17 cells. Th17 cells subsequently release IL-17 and IL-22, which promote neutrophil recruitment to infection sites. On the other hand, *N. gonorrhoeae* inhibits antigen-presenting cell secretion of IL-12 for Th1 differentiation and IL-4 for Th2 differentiation. Therefore, the production of Th1-derived interferon (IFN)-γ and Immunoglobulin (Ig)G2a, as well as Th2-derived IL-4, IL-5 and IgG1, is suppressed. *N. gonorrhoeae* also induces type 1 regulatory T (Treg) cells, which produce IL-10 and inhibit Th1, Th2 and Th17 responses. Image created using BioRender (https://www.biorender.com).

In murine models, infection with *N. gonorrhoeae* also guides responses towards a Th17-dominated profile, suppressing Th1-driven memory responses and so inhibiting long-term immune memory following infection^40,41^ (Fig. 2). Reshaping the cytokine milieu to favour Th1 responses with treatment of IL-12 (pro-Th1)^42^ or anti-IL-10 antibody (anti-Treg)^43^ during primary infection, has been shown to enhance serum immunoglobulin (Ig) G responses to infection and promote bacterial clearance in murine models of genital gonorrhoea.

Overall, *N. gonorrhoeae* has evolved various strategies to evade both innate and adaptive immune responses, especially by interfering with complement activation and manipulating cellular immunity. These mechanisms contribute to persistent colonisation and frequent reinfections. Importantly, these insights highlight three key considerations for vaccine development: the extensive diversity in antigens expressed by *N. gonorrhoeae*, its ability to subvert immune responses, and the need to induce Th1-driven responses to establish durable protective immunity.

## Meningococcal OMV vaccines: evidence of cross-protection

Despite longstanding challenges in *N. gonorrhoeae* vaccine development, in 2017, the field was reinvigorated by the unexpected retrospective observation that the MeNZB vaccine was associated with reduced incidence of symptomatic *N. gonorrhoeae* infection. Initially deployed to curb an outbreak caused by serogroup B *N. meningitidis* NZ98/254 strain in New Zealand, the MeNZB vaccine consists of the OMVs from this strain adjuvated with aluminium hydroxide^44^. The retrospective post-hoc analysis showed that MeNZB conferred 31% vaccine effectiveness against gonorrhoea in individuals aged 15–30 years that had received the MeNZB vaccine^5^. This cross-protection likely stemmed from the close genetic, and hence antigenic, relatedness of *N. meningitidis* and *N. gonorrhoeae*, and the polyvalent nature of antigens in the MeNZB OMV. The study highlighted the potential of using OMV-based vaccines for the prevention of gonorrhoea.

OMVs are spherical 20–250 nm diameter vesicles shed from the outer membrane of Gram-negative bacteria, including *Neisseria* spp.^45^. These vesicles contain periplasmic components, outer membrane proteins, LOS, lipids and lipoproteins, and so consist of a rich diversity of antigens with immunogenic potential^45^. The broad antigen repertoire of OMVs in MeNZB provides a biologically plausible explanation for their potential cross-protection against *N. gonorrhoeae*.

Another licensed *N. meningitidis* vaccine, 4CMenB (Bexsero, GSK), contains the same OMVs as MeNZB. Additionally, 4CMenB contains three recombinant *N. meningitidis* antigens: factor H-binding protein (fHbp) fused to a *Neisseria* antigen, GNA2091, *Neisseria* heparin-binding antigen (NHBA) fused to GNA1030, and *Neisseria* adhesin A (NadA)^46^. Of these, only NHBA is expressed on the surface of *N. gonorrhoeae*^47^. Consistent with the New Zealand study, observational studies after the introduction of 4CMenB in the general populations of the United States^6,7,9^ and Australia^8^ also indicate a modest degree of cross-protection against *N. gonorrhoeae* (23-46% reduction).

## 4CMenB-induced immune responses: of men and mice

Following the encouraging evidence regarding MeNZB and 4CMenB, there has been considerable interest in assessing their immunogenicity against *N. gonorrhoeae* in clinical studies and in animals, particularly their ability to protect against challenge in the murine female genital tract model. Efforts have also been made to identify the antigens recognised by 4CMenB-induced serum antibodies using a number of *N. gonorrhoeae* strains, including FA1090 and 1291^48^, as well as F62, FA19, MS11 and WHO X^49^ (Fig. 1), with the aim of defining vaccine candidates.

The protective efficacy of 4CMenB has been consistently recapitulated in the murine model^50–52^. This is a significant step forward for the field, as it reinforces the value of this model for the preclinical evaluation of *N. gonorrhoeae* vaccine candidates. To facilitate colonisation by human-adapted *N. gonorrhoeae*, female mice are implanted with a 17β-estradiol pellet prior to infection, which maintains the vaginal epithelium in a prolonged oestrous-like state^53^. At the same time, mice are treated with broad-spectrum antibiotics to deplete the commensal microbiota of the genital tract, thereby reducing competition for ecological niches and nutrients. Together, these modifications enable consistent murine vaginal colonisation by *N. gonorrhoeae*, typically lasting up to 10 to 12 days.

### Antibody responses against OMVs target diverse *N. gonorrhoeae* antigens

Several studies have identified the individual antigens that contribute to the cross-species immunogenicity of 4CMenB and the characterisation of the types of antibodies induced. A murine study found that subcutaneous or intraperitoneal administration of 4CMenB induced IgG responses against several *N. gonorrhoeae* antigens, including Opa, PorB, PilQ (the outer membrane secretin needed for pilus biogenesis), BamA (involved in inserting proteins in the outer membrane), and MtrE (the surface component of a multidrug efflux pump)^50^.

**Fig. 3:**
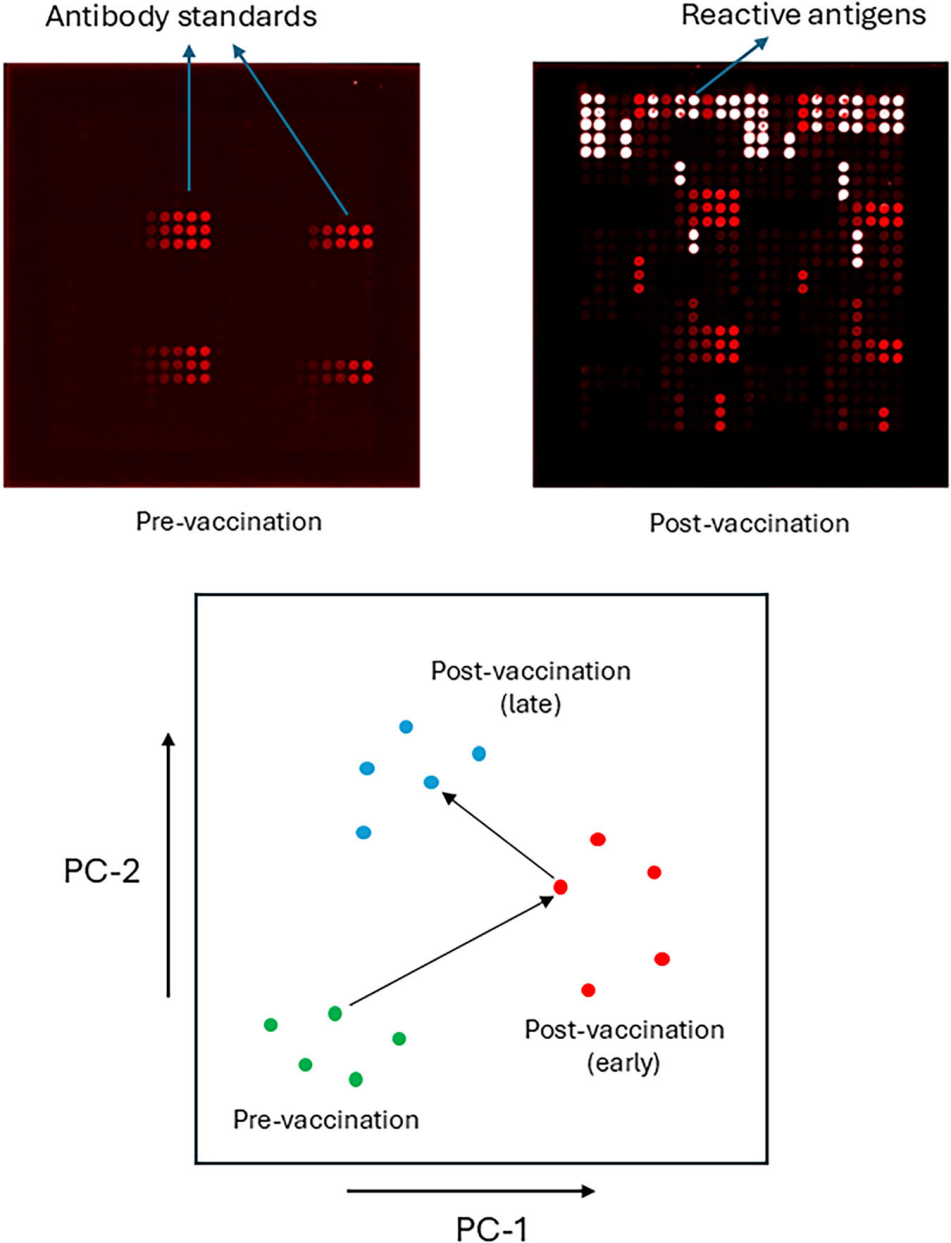
Application of principal component analysis (PCA) to antigen microarray data. The method uses a whole dataset approach, rather than considering antigen responses singly. *Upper panel:* example raw data; each spot is a calibrated quantity of antigen printed onto the array. Responses to multiple individual antigens are apparent following vaccination and are quantified by fluorescence intensity. The example is from murine data which exhibit low pre-vaccination backgrounds; human serum frequently harbours antibodies against specific antigens even before vaccination^55,93^. *Lower panel:* schematic illustration of the application of PCA multidimensional data reduction. Each point is a serum sample; points close in space signify similar profiles of antigen reactivities. Serial samples taken from the same individual can be followed (example with black arrows).

Consistent with these findings, a clinical study assessed responses to 4CMenB administered to high-risk individuals in Kenya using a *N. gonorrhoeae* antigen microarray based on the strain FA1090; findings confirmed that 4CMenB induces antibodies against multiple *N. gonorrhoeae* antigens, including PilQ, membrane protein 2 and NHBA^54^. Reduction of antigen reactivity datasets generated from antigen microarrays can be used to chart the complex polyclonal antibody responses to OMV-based vaccines in vaccinated populations (Fig. 3)^54,55^. A separate study noted that approximately 10% of IgG in low-risk individuals recognised diverse epitopes in *N. gonorrhoeae* LOS^56^, with anti-LOS antibodies synergising with anti-NHBA antibodies to produce potent serum bactericidal activity^47^. A further study characterised human monoclonal antibodies (mAbs) elicited by 4CMenB by cloning B cells from vaccine recipients ^57^. Interestingly, all the mAbs reported to date target either PorB or LOS, which are highly variable *N. gonorrhoeae* antigens. The limited repertoire of mAbs was surprising, given the breadth of anti-*N. gonorrhoeae* polyclonal antibody responses seen in other human studies and in immunised mice, but may highlight the immunodominance of PorB and LOS as targets of human antibody responses.

### Antibody isotypes: the roles of IgG and IgA

The class of antibody elicited by vaccination is a crucial factor for understanding the potential cross-species immunity induced by 4CMenB. For effective protection against *N. gonorrhoeae*, which tends to colonise mucosal surfaces, both serum IgG and mucosal IgA responses are likely to be required^58,59^.

In female mice, immunisation with 4CMenB induced serum and vaginal IgG, as well as vaginal IgA, and provided protection against challenge with *N. gonorrhoeae* F62^50^. In humans, 4CMenB induced both serum IgG and IgA; interestingly, responses of both antibody classes to the recombinant protein components of 4CMenB peaked at 10 weeks after immunisation but had declined by 24 weeks. Outer membrane proteins from the OMV component of the vaccine, such as BamA, tended to show stronger antibody reactivities at the 24-week time point^54^. In a separate study, murine and human antibody responses were compared using a multiplexed bead-based assay; this can quantify IgG and IgA simultaneously, using 70-fold less antigen than the traditional enzyme-linked immunosorbent assay (ELISA)^60^. Preliminary results suggest that 4CMenB in humans induces serum IgG against PorB and MtrE, as well as IgA against PorB^60^.

### Understanding correlates of protection

Given the concordance between human and murine data, recent work has attempted to define the correlates of 4CMenB protection in the murine model by examining immune responses and comparing them with the extent and duration of infection in individual animals.

One study examined how 4CMenB and an engineered OMV from *N. meningitidis* (MC58 Δ*porA* Δ*porB* Δ*rmpM*) protect against *N. gonorrhoeae* infection in the female mouse genital tract model^52^. Compared to adjuvant-only controls, both vaccines significantly induced elevated serum and vaginal anti-*N. gonorrhoeae* antibodies, as well as splenocyte cytokine responses (e.g. IFN-γ, IL-17), and also accelerated *N. gonorrhoeae* clearance. Notably, the immunologic parameters linked to lower bacterial burden differed between the two vaccines, indicating that each may rely on distinct combinations of humoral and cellular immunity.

In another study, 4CMenB-immunised mice were categorised as the ‘protected’ or ‘not protected’ group based on whether they cleared *N. gonorrhoeae* infection by day 3 following a vaginal challenge^51^. Post-challenge analysis of cellular responses showed that subcutaneous administration of 4CMenB elicited multifaceted T-cell responses spanning Th1, Th2, Th17 and Treg subsets, both systemically and within the genital tract. Despite this robust cellular activation, neither T‑cell profiles nor splenocyte cytokines differed significantly between protected mice and non-protected ones. Instead, mucosal non‑T and non‑B lymphocyte populations were highlighted as potential mediators of protection, underscoring the complexity of identifying effective immune correlates.

To date, research has investigated the ability of 4CMenB to induce immune responses against *N. gonorrhoeae* in both human and murine models. Studies show that 4CMenB vaccination triggers both systemic IgG and mucosal IgA antibodies targeting multiple *N. gonorrhoeae* antigens, alongside cellular immune responses. While murine models confirm vaccine efficacy and help identify immune correlates, these correlates are complex, not reliant on a single immunologic marker, and may vary between vaccine formulations.

## Novel avenues for OMV vaccines against *N. gonorrhoeae*

Building on the serendipitous observations from *N. meningitidis* OMV vaccines, several strategies have been proposed for developing OMV-based vaccines against *N. gonorrhoeae*. One key consideration is the method used for OMV extraction. Because native OMVs are present at low abundance, large-scale OMV production often requires extraction from bacteria with detergents (e.g. sodium deoxycholate), as used for MeNZB and 4CMenB^44,46^. Detergent treatment offers both advantages and disadvantages; it can reduce the endotoxicity associated with lipid A^61^, but can also deplete potentially immunogenic lipoproteins^62^. Other equally important factors include the OMV-producing strain (e.g*. N. meningitidis*, *N. gonorrhoeae*, or commensal *Neisseria* spp.), which is used to classify the vaccine approaches discussed in the following sections, as well as the choice of OMV adjuvant and administration route (Table 2).

**Table 2:** OMV vaccines against *N. gonorrhoeae*

Vaccine design			Vaccine-induced immunity				Refs.
OMV-producing strain	Detergent treatment	Adjuvant	Animal model (administration route)	Immune responses specific to *N. gonorrhoeae* after vaccination	Serum bactericidal activity against *N. gonorrhoeae*	Protection from *N. gonorrhoeae* challenge	
*N. meningitidis* OMV vaccines							
*N. meningitidis* B:4:P1.7b,4 (4CMenB)	Deoxycholate	Aluminium hydroxide	Human (intramuscular)	Serum antibodies against 1291 whole cell	/	/	^48^
			Rabbit	Serum antibodies against FA1090 and 1291 whole cell lysates	/	/	^48^
			Mouse (subcutaneous)	Serum antibodies against FA1090, F62, FA19, MS11 and WHO X whole cells	FA1090	/	^49^
			Mouse (intraperitoneal or subcutaneous)	Serum and vaginal IgG (but not IgA) against F62 OMVs Serum antibodies against PilQ, BamA, MtrE, PorB and Opa	F62 FA1090	F62 (in 17β-estradiol-treated female mice)	^50^
			Human (intramuscular)	/	Not for F62	/	
			Human (intramuscular)	Serum IgG against NHBA, RmpM, BamA, membrane protein 2 and PilQ Serum IgA against NHBA, RmpM and membrane protein 2	/	/	^54^
			Human (intramuscular)	Serum antibodies against LOS of FA19 and F62	/	/	^56^
			Human (intramuscular)	NHBA antibodies are essential and synergise with LOS antibodies in serum bactericidal activity against 1291	/	/	^47^
			Human (intramuscular)	Serum monoclonal antibodies against PorB and LOS	/	/	^57^
			Human (intramuscular)	Serum IgG against PorB and MtrE Serum IgA against PorB	/	/	^60^
*N. meningitidis* MC58 Δ*porA* Δ*porB* Δ*rmpM*	Sodium deoxycholate	Alhydrogel	Mouse (intraperitoneal)	Serum IgG against MtrE and PilQ Serum and vaginal IgG1, IgG2a, IgG2b, and IgA against F62 whole cell	F62 and MS11, but not FA1090	F62	^64,66^
*N. meningitidis* serogroup B H44/76 Δ*lpxl1*	No detergent	Alhydrogel	Mouse (intraperitoneal)	/	FA1090	/	^67^
*N. gonorrhoeae* OMV vaccines							
*N. gonorrhoeae* MS11	No detergent	No adjuvant	Mouse (intranasal)	Serum antibodies and vaginal IgA and IgG against MS11 OMVs	MS11	MS11	^68^
*N. gonorrhoeae* FA1090 Δ*rmpM*	No detergent	No adjuvant	Mouse (intranasal)	Serum IgG and vaginal IgG and IgA against PorB IFN-γ response of splenocytes restimulated with PorB	/	/	^69^
*N. gonorrhoeae* FA1090	No detergent	IL-12 encapsulated in poly-lactic acid microspheres	Mouse (intravaginal)	Serum and vaginal IgG and IgA against FA1090 whole cell IFN-γ response of iliac lymph node cells restimulated with FA1090 OMVs plus IL-12	/	FA1090, MS11 and FA19	^75^
*N. gonorrhoeae* MS11			Mouse (intravaginal)	/	/	FA1090	
*N. gonorrhoeae* FA19			Mouse (intravaginal)	/	/	FA1090	
*N. gonorrhoeae* FA1090 (wild-type is more effective than Δ*lpxL1*)	No detergent (more effective than deoxycholate)	IL-12 encapsulated in poly-lactic acid microspheres	Mouse (intranasal)	Serum IgG and vaginal IgG and IgA against WHO F, WHO L and WHO W whole cells	/	FA1090, WHO F and WHO W	^76^
*N. gonorrhoeae* FA1090 _*Nm* MC58 PorB_ Δ*rmpM*	No detergent	No adjuvant	Mouse (intraperitoneal)	Higher serum IgG2a (Th1 response) / IgG1 (Th2 response) ratio against MtrE and MetQ IFN-γ response of splenocytes restimulated with engineered OMVs	FA1090, 60755	/	^77^
Commensal *Neisseria* OMV vaccines							
*N. lactamica*	Not known	Alhydrogel	Mouse (subcutaneous or intranasal)	/	/	/	^79^
*N. cinerea* ATCC 14685 _*Ng* NHBA_ Δ*rmpM* Δ*fHbp*	No detergent	Alhydrogel	Mouse (intraperitoneal)	Serum antibodies against FA1090 and piliated 1291 whole cell lysates	/	/	^80^

### Enhancing OMV vaccines derived from *N. meningitidis*

Following 4CMenB, new *N. meningitidis* OMV vaccines targeting *N. gonorrhoeae* have been explored. One such vaccine contains OMVs extracted using the detergent, sodium deoxycholate, from *N. meningitidis* MC58 Δ*porA* Δ*porB* Δ*rmpM*, with additional adjuvant Alhydrogel^63,64^. These genetic deletions were made because PorA is not expressed by *N. gonorrhoeae*, PorB exhibits high variability^65^, and RmpM induces blocking antibodies. In murine models, intraperitoneal administration of the vaccine induced serum IgG against MtrE and PilQ, and serum bactericidal activity against serum-sensitive *N. gonorrhoeae* F62 and MS11, though not the serum-resistant strain, FA1090^64^. The vaccine also accelerated the clearance of *N. gonorrhoeae* F62 in the 17β-estradiol treated female murine model compared with OMVs generated from wild-type *N. meningitidis*; however, the difference was not seen consistently in a second trial^64^. A more recent study confirmed that the Δ*porA* Δ*porB* Δ*rmpM* OMV candidate vaccine promoted clearance of the gonococcus from the murine reproductive tract, following subcutaneous or intraperitoneal vaccination. Antigen microarray analysis showed IgG reactivities against a range of antigens, including the type IV pilus secretin PilQ^66^.

Vaccines developed with native *N. meningitidis* OMVs, preserving most membrane proteins^62^, have also shown promise. A vaccine was developed containing native OMVs generated from *N. meningitidis* serogroup B H44/76 Δ*lpxl1* and the adjuvant Alhydrogel^67^. The *lpxL1* gene is responsible for the addition of an acyl chain to lipid A, and its removal reduces the potency of bacterial endotoxin without the need for detergent extraction^67^. Intraperitoneal administration of this vaccine to mice induced serum bactericidal activity against FA1090^67^.

### Development of *N. gonorrhoeae*-specific OMV vaccines

Before the discovery of 4CMenB’s cross-protection against *N. gonorrhoeae*, several studies used the murine model to explore the protective efficacy of OMV vaccines derived directly from *N. gonorrhoeae*. Early findings showed that intranasal immunisation with EDTA-extracted OMVs generated from *N. gonorrhoeae* strain MS11 induced serum with bactericidal activity and accelerated the clearance of MS11 in 17β-estradiol treated female mice^68^. Another study in mice found that OMVs generated from *N. gonorrhoeae* FA1090 Δ*rmpM* (to eliminate blocking antibody responses against RmpM) induced both serum and vaginal antibody responses, and increased IFN-γ production from immune splenocytes upon PorB restimulation, indicating a shift towards Th1 responses^69^. Other studies defining cellular immune responses also revealed that *N. gonorrhoeae* OMVs activated the pro-inflammatory transcriptional factor, nuclear factor kappa B (NF-κB)^70^ and promoted CD4^+^ T cell proliferation^71^. Despite these promising findings, disappointingly, immunisation with *N. gonorrhoeae* OMVs did not provide any demonstrable or reproducible protection against *N. gonorrhoeae* challenge^72^.

The lack of protection by *N. gonorrhoeae* OMVs could be due to differences between *N. gonorrhoeae* and *N. meningitidis*, especially in PorB and RmpM. As already mentioned, *N. gonorrhoeae* PorB is highly diverse and inhibits complement pathways by recruiting the human complement inhibitors, factor H^30^ and C4b-binding protein^29^. *N. gonorrhoeae* PorB also inhibits dendritic cell-stimulated CD4^+^ T cell proliferation^33^. In contrast, there is evidence that *N. meningitidis* PorB stimulates broad B and T cell responses in mice, with elevated levels of IgG1 (indicating Th2 responses), IgG2b (Th1 responses), and a robust IFN-γ^+^ CD4^+^ and IFN-γ^+^ CD8^+^ T cell response^73^. Similarly, *N. gonorrhoeae* RmpM induces blocking antibodies^31^, while *N. meningitidis* RmpM does not appear to have such effects^74^. These divergent immune properties likely contribute to the failure of *N. gonorrhoeae*-derived OMV vaccines to induce lasting protection.

More recent studies have focused on improving the immunogenicity of *N. gonorrhoeae* OMV vaccines, by specifically enhancing Th1 responses to promote immunological memory and addressing the challenges of a highly variable repertoire of surface antigens.

### Optimisation of adjuvants, OMV extraction and vaccination route

One approach involves the use of the cytokine IL-12 as an adjuvant. IL-12 is known to reshape the immune milieu towards Th1 responses and enhances adaptive immune memory against *N. gonorrhoeae*^42^. A vaccine was developed that combines detergent-free OMVs generated from *N. gonorrhoeae* FA1090 with IL-12 encapsulated in poly-lactic acid microspheres^75^. A murine study showed that intravaginal administration of this vaccine induced higher levels of serum and vaginal IgG and IgA than OMVs without IL-12, and also higher IFN-γ production from CD4^+^ and CD8^+^ T cells in iliac lymph nodes upon restimulation by the vaccine^75^. Moreover, this vaccine provided enhanced protection against challenge by not only homologous (FA1090) but also heterologous (MS11, FA19) strains^75^, indicative of broad protective efficacy.

The effects of vaccination routes and OMV extraction methods were subsequently investigated. Murine studies showed that intranasal administration of the FA1090 OMV + IL-12 vaccine induced higher levels of serum, vaginal and saliva antibodies compared with intravaginal administration, and accelerated the clearance of *N. gonorrhoeae* following challenge^76^. These results suggest that intranasal vaccination may provide an effective and more feasible alternative route for human immunisation. Regarding OMV extraction, treatment with the detergent deoxycholate reduced serum IgG and vaginal IgA levels after immunisation, although it did not affect markers of Th1 responses, such as IFN-γ production by CD4^+^ T cells in iliac lymph nodes, or indeed the clearance of *N. gonorrhoeae*^76^. This finding indicates that detergent-free isolation of OMVs might enhance immune responses.

### Attempts to circumvent gonococcal immune evasion

Another approach for improving *N. gonorrhoeae* OMV vaccines involves genetically modifying *porB* and *rmpM* to impair their ability to mediate immune evasion. Detergent-free OMVs were generated from *N. gonorrhoeae* strain FA1090, in which the immunosuppressive PorB was exchanged with PorB from *N. meningitidis* MC58, and *rmpM* was deleted to prevent the development of blocking antibodies^77^. A murine study showed that intraperitoneal immunisation with OMVs from this modified strain induced a higher ratio of serum IgG2a (Th1 response) to IgG1 (Th2 response) against MtrE and the methionine transporter MetQ, and increased IFN-γ production (Th1 response) by splenocytes restimulated by this engineered OMV, when compared with OMVs from *N. gonorrhoeae* FA1090 Δ*rmpM* with native PorB, indicating a Th1-biased immune response^77^.

Proteomic analyses revealed that OMVs from the *N. gonorrhoeae* strain with the *N. meningitidis* PorB contained higher levels of several virulence-associated antigens, including Opa, MetQ, NHBA and MtrE, compared with those from the strain with the native *N. gonorrhoeae* PorB^77^. This observation suggests that genetic modifications can be employed to enhance the immunogenic profile of OMVs. Moreover, antigen profiling of these OMVs closely matched the proteomic profiles of clinical isolates from the pharynx, cervix, vagina and urethra^78^.

In summary, recent advancements in *N. gonorrhoeae* OMV vaccines, which either incorporate IL-12 microspheres to boost Th1 responses or genetic modification of *porB* and *rmpM*, have demonstrated enhanced immunogenicity and promising protection in mice. These strategies present promising approaches for developing more effective OMV vaccines based on *N. gonorrhoeae* itself.

### Commensal *Neisseria* OMV platforms

In addition to *N. meningitidis* and *N. gonorrhoeae*, commensal *Neisseria* species have been explored as a safer vaccine platform. For example, subcutaneous or intranasal immunisation of OMVs from *Neisseria lactamica*, a commensal of the human nasopharynx, has been found to act as an effective adjuvant by inducing serum IgG and nasal-associated lymphoid tissue IgA responses^79^. Interestingly, the induced serum IgG recognise *N. meningitidis* OMVs, similar to the cross-protective effects observed with *N. meningitidis* OMVs against *N. gonorrhoeae*.

Alternatively, OMVs from *Neisseria cinerea*, a non-pathogenic species of the oropharynx, have been investigated in combination with the adjuvant Alhydrogel^80^. *N. cinerea* was genetically modified to remove RmpM, delete fHbp (which is not surface exposed in *N. gonorrhoeae)*, and express *N. gonorrhoeae* NHBA. Intraperitoneal administration of this vaccine to mice induced serum antibodies that cross-react with *N. gonorrhoeae* antigens.

These findings highlight the potential of commensal *Neisseria* OMVs as a safe platform for developing vaccines against *N. gonorrhoeae* and *N. meningitidis*. Moreover, *Escherichia coli* OMVs have been explored as natural vaccine carriers for heterologous antigens^81^, further illustrating the versatility of OMVs as a platform for vaccine development.

## Conclusions and perspectives

The development of vaccines against *N. gonorrhoeae* has been challenging due to extensive HGT, diversity in surface-exposed antigens, and the capacity of *N. gonorrhoeae* to subvert innate and adaptive immunity, such that natural infection does not induce immunological memory and protection. However, there has been substantial activity in the field since observational studies demonstrated that immunisation with *N. meningitidis* OMVs is associated with reduced incidence of *N. gonorrhoeae* infection. Researchers have subsequently been trying to detail 4CMenB’s immunogenicity against *N. gonorrhoeae*, with the aim to further enhance immune responses by developing improved OMV-based vaccines derived from *N. meningitidis*, *N. gonorrhoeae* or commensal *Neisseria*. These vaccines incorporate various genetic modifications, extraction methods, adjuvants and administration routes (Table 2).

However, certain caveats remain about the efficacy of the potential use of *N. meningitidis* OMVs as a *N. gonorrhoeae* vaccine. Notably, in the New Zealand MeNZB study, there was no evidence for cross-protection against *N. gonorrhoeae* in individuals co-infected with *Chlamydia trachomatis*, another prevalent STI^5^. A potential explanation for this result is that *C. trachomatis* modulates the local immune environment and interferes with OMV-induced responses. Additionally, any protection provided by 4CMenB seems limited in high-risk populations. A study in France reported an adjusted hazard ratio of 0.78 for gonorrhoea incidence in vaccinated versus unvaccinated individuals, close to 1, indicating no significant protection^82^. This result may be influenced by the high frequency of prior *N. gonorrhoeae* infection amongst study participants (men who have sex with men), the use of post-exposure prophylaxis, and the prevalence of tetracycline-resistant isolates.

Nevertheless, the potential to confer cross-protection against *N. gonorrhoeae* remains a promising direction for OMV-based vaccines. Ongoing clinical trials are evaluating the efficacy of 4CMenB against *N. gonorrhoeae*^83^, and the UK Health Security Agency has started the 4CMenB programme for use in high-risk populations^10^. The vaccine will be offered to individuals at higher risk of gonorrhoea, including those with a recent *N. gonorrhoeae* infection or other bacterial STIs, with multiple sexual partners, or if they belong to key populations. This will provide further opportunity to investigate the extent and correlates of any potential benefit of 4CMenB in a target group. In addition to more clinical trials of *N. meningitidis* OMV vaccine 4CMenB, a phase 2 clinical trial of a GSK *N. gonorrhoeae* OMV vaccine derived from FA1090Δ*rmpM*Δ*lpxL1* ^84,85^ reportedly failed to meet its pre-defined efficacy criteria^86^. Full results are eagerly anticipated by the research community, as the outcome could offer insights into better ways to design vaccines tailored for the prevention of *N. gonorrhoeae* disease.

While there is encouraging evidence of cross-protection with heterologous OMVs (i.e. derived from a different species), it is likely that more successful approaches will apply OMVs from *N. gonorrhoeae* to protect against gonorrhoea. In the future, research on OMV vaccines against *N. gonorrhoeae* should focus on several areas: (1) thorough consideration of the *N. gonorrhoeae* population structure to inform strain selection; (2) comprehensive characterisation of the dominant immunogenic antigens in current OMV vaccines through proteomic analysis; (3) a deeper understanding of *N. gonorrhoeae* pathogenesis to guide the genetic modification of antigens in OMV-producing bacteria, such as the deletion of immunosuppressive genes or overexpression of immunostimulatory genes, and to inform OMV adjuvant choices; and (4) prospective immunology studies to evaluate how antibody and cellular responses protect against *N. gonorrhoeae* in immunised individuals, taking into consideration any differences between males and females. Ultimately, the development of effective OMV vaccines against *N. gonorrhoeae* has the potential to significantly reduce gonorrhoea and improve sexual and reproductive health worldwide.

## Data Availability

No datasets were generated or analysed during the current study.
